# Examination of relaxin and its receptors expression in pig gametes and embryos

**DOI:** 10.1186/1477-7827-9-10

**Published:** 2011-01-20

**Authors:** Jean M Feugang, Juan C Rodriguez-Munoz, Scott T Willard, Ross A Bathgate, Peter L Ryan

**Affiliations:** 1 Department of Animal & Dairy Sciences, Mississippi State University, 4025 Wise Center, Mississippi State, MS 38762, USA; 2 Department of Biochemistry & Molecular Biology, Mississippi State University, 402 Dorman Hall, Mississippi, MS 38762, USA; 3 Florey Neuroscience Institutes, University of Melbourne, Gate 11, Royal Parade, Victoria, 3010, Australia; 4 Department of Pathobiology & Population Medicine, Mississippi State University, 240 Wise Center Dr., Mississippi State, MS 38762, USA

## Abstract

**Background:**

Relaxin is a small peptide also known as pregnancy hormone in many mammals. It is synthesized by both male and female tissues, and its secretions are found in various body fluids such as plasma serum, ovarian follicular fluid, utero-oviduct secretions, and seminal plasma of many mammals, including pigs. However, the presence and effects of relaxin in porcine gametes and embryos are still not well-known. The purpose of this study was to assess the presence of relaxin and its receptors RXFP1 and RXFP2 in pig gametes and embryos.

**Methods:**

Immature cumulus-oocyte complexes (COCs) were aspirated from sows' ovaries collected at the abattoir. After *in vitro*-maturation, COCs were *in vitro*-fertilized and cultured. For studies, immature and mature COCs were separately collected, and oocytes were freed from their surrounding cumulus cells. Denuded oocytes, cumulus cells, mature boar spermatozoa, zygotes, and embryos (cleaved and blastocysts) were harvested for temporal and spatial gene expression studies. Sections of ovary, granulosa and neonatal porcine uterine cells were also collected to use as controls.

**Results:**

Using both semi-quantitative and quantitative PCRs, relaxin transcripts were not detected in all tested samples, while RXFP1 and RXFP2 mRNA were present. Both receptor gene products were found at higher levels in oocytes compared to cumulus cells, irrespective of the maturation time. Cleaved-embryos contained higher levels of RXFP2 mRNA, whereas, blastocysts were characterized by a higher RXFP1 mRNA content. Using western-immunoblotting or *in situ *immunofluorescence, relaxin and its receptor proteins were detected in all samples. Their fluorescence intensities were consistently more important in mature oocytes than immature ones. The RXFP1 and RXFP2 signal intensities were mostly located in the plasma membrane region, while the relaxin ones appeared homogeneously distributed within the oocytes and embryonic cells. Furthermore, spermatozoa displayed stronger RXFP2 signal than RXFP1 after western-immunoblotting.

**Conclusion:**

All together, our findings suggest potential roles of relaxin and its receptors during oocyte maturation, early embryo development, and beyond.

## Background

The inadequate culture conditions greatly limit the production of high quality embryos [1,2]. *In vivo*, maturing gametes and developing embryos maintain complex interactions with their immediate environments which are rich in a variety of molecules such as relaxin, whose embryotrope effects are not completely understood [3,4]. Relaxin is a small peptide (≈ 6 kDa) commonly known as a pregnancy hormone in many mammals [5,6], consisting of several members expressed in various tissues across a broad range of mammalian species [6,7]. Consequently, relaxin is found in a variety of body fluids and has pleiotropic actions on numerous tissue targets [6,8]. In female reproductive tissues, relaxin is involved in a range of events such as ovarian follicle growth and ovulation, development of mammary glands, preparation of the uterus and cervix for pregnancy and delivery, while relaxin's action in males is mainly limited to the improvement of sperm motility [3,6,8].

These various effects of relaxin are mediated through a family of plasma membrane receptors known as RXFP1, 2, 3 and 4 [9]. Ovarian relaxin or relaxin-2 is the specific ligand of RXFP1 (or LGR7), but also binds with low affinity to RXFP2 (or LGR8), the natural receptor of insulin-like peptide 3 (INSL3) [10]. Different molecular and immunological techniques have been used to detect their expression in mammalian tissues [11-13], including oocytes of primates [11,12] and rats [14].

Despite the high relaxin levels found in follicular fluids and oviduct environment of sows, there are still no reports available on the expression of these receptors in porcine oocytes and embryos [15-20]. This presence of relaxin may suggest its potential roles during oocyte developmental competency acquisition and early embryo development. Indeed, relaxin detection in follicular fluids and granulosa cells has been purposed as a predictor of successful embryo transfer in humans and early pregnancy status in common marmosets [21,22].

From this background, the present study aimed at investigate the possible expressions of relaxin and its receptors RXFP1 and RXFP2 in porcine gametes and cultured embryos using both semi-quantitative and quantitative PCR techniques, western-immunoblotting and *in situ *immunofluorescence approaches.

## Methods

### Chemicals and media

Unless otherwise indicated, all chemicals and reagents were purchased from Sigma-Aldrich (Saint Louis, USA) for embryo production or Invitrogen Co. (Carlsbag, USA) for gene expression. Relaxin (pRLN) obtained from pregnant sow ovaries was a gift from Dr. C Bagnell laboratory [23]. INSL3 was purchased from Phoenix Pharmaceutics, Inc. (Burlingame, USA), respectively. Ovaries were washed in NaCl (0.9%; w/v) and oocytes and embryos in Hepes-buffered Tyrode Lactate medium supplemented with polyvinyl alcohol (PVA; 0.1%; w/v) and pyruvate (100 μM). Cumulus-oocyte complexes were matured in TCM199+L-glutamine medium supplemented with PVA (1%; w/v), glucose (2.8 mM), pyruvate (0.91 mM), cysteamine (0.57 mM), EGF (10 ng/mL), and FSH (0.4 μg/mL), and fertilized in the modified Tris-buffered medium containing caffeine (2 mM) and BSA-fraction V (0.1%; w/v). Embryos were cultured in NCSU-23 medium supplemented with BSA-FAF (0.4%; w/v). All media contained 10 μl/mL penicillin/streptomycin and were pre-incubated at 37°C for at least 2 h before use.

### Cumulus-oocyte complexes (COCs) collection and in vitro embryo production

Sow (Yorkshire-Landrace) ovaries were collected at a local abattoir (Southern Quality Meats, Pontotoc, USA) and transported at 37°C to the laboratory. Immature COCs were aspirated from medium size follicles (3-6 mm of diameter), washed and *in vitro*-matured (50-75 COCs per 500 μl) for 44 hours. Diluted (in BTS) pools semen of at least two boars were purchased (Prestage Farms; West Point, MS, USA) and high motile spermatozoa were purified by centrifugation through a percoll gradient (90%:45%). Final concentrations of 6 × 10^5 ^spermatozoa/ml were used to fertilize the COCs (= 0 h post-insemination or 0 hpi). After 18 hpi, presumptive zygotes were harvested, mechanically denuded and cultured (1 embryo/1-2 μl of culture medium) for up to 6 days (Day 7pi). All incubations took place in a humidified atmosphere of 39°C and 5% CO_2 _in air.

In each experimental replicates, groups of oocytes were treated separately as controls for embryo production. The current culture system allowed maturation of approximately 70% oocytes, formation of 50% zygotes after fertilization, cleavage of 42% ± 4.5% embryos, and development of 10% ± 1.7% blastocysts. All proportions were calculated upon the total number of COCs placed in maturation.

### Sample collection for gene expression

Oocytes and embryos were selected under the stereomicroscope based on their morphology and cytoplasm homogeneity. Groups of 10 immature and mature COCs were collected, and oocytes were mechanically separated from their corresponding cumulus cells. Groups of 10 zygotes (18 hpi), cleaved-embryos (2-4 cells; Day 2 pi) were collected and pooled separately, while blastocysts (Day 7pi) were collected in pools of three. In parallel, subsets of high motile spermatozoa, neonate porcine uterine cells (Ut), mural granulosa cells (GC), and sections of sow ovarian corpus luteum (CL) were collected. All samples were obtained from at least three independent repeats and stored at -80°C for total RNA and protein isolations.

### RNA isolation and RT-PCR

Total RNA of all frozen-thawed samples were isolated (RNeasy Micro kit; Qiagen Inc., Valencia, CA, USA) and reverse-transcribed into cDNA (Superscript III Platinum^® ^Two-Step qRT-PCR Kit) used for both semi-quantitative (Taq DNA polymerase kit) and real-time (SYBR^® ^GreenER™ qPCR SuperMixes for iCycler) PCRs. The expression of relaxin, RXFP1, RXFP2, and β-actin (used as internal control) genes was assessed using the following PCR conditions: 5 min at 95°C; 45 cycles of [30 sec at 95°C, 30 sec at the optimal annealing temperature (Table 1) and 30 sec at 72°C]; 10 min at 72°C. Real-time PCRs were performed and the comparative Ct method was used to determine the transcript levels as previously described [24]. Amplification products were resolved on 1.5% agarose gels. Porcine neonatal uterine cells and corpus luteum total RNA were used as positive controls after verification of PCR product authenticities by sequencing (BigDye Terminator V1.1 cycle sequencing kit; Applied Biosciences Inc., Foster City, CA, USA) and BLAST on pig genome (NCBI repository database).

**Table 1:** Porcine primer pair characteristics

Gene names	GenBank Acc. (NCBI)	Primer sequences (5'-3')	**AT (**^**o**^**C)**	Amplicon sizes (bp)
RXFP1	CA994862.1	S: AGGCTGACGAGGACAACT	52.5	132
		AS: CAGAACCGACCAAGCATT		
RXFP2	CA997681.1	S: CATCTGCTGGATTCCCGTAT	55	117
		AS: TTCAAGGCACTGTTCACC		
RELAXIN	NM213872.1	S: TGTGGCTCCGTCTCCTGGGG	55	164
		AS: GTTGCCTTCAGCTCCTGTGGC		
β-ACTIN	U07786	S: 5'-ACTGGCATTGTCATGGACTCTG-3'	60	397
		AS: 5'-AGTTGAAGGTGGTCTCGTGGAT-3'		

AT = Annealing temperature

### Protein isolation and analyses

#### Selection of antibodies

Anti-porcine relaxin and RXFP1 and RXFP2 antibodies are not commercially available. Therefore, we used the anti-human ones (Santa Cruz Biotech Inc. Santa Cruz, CA, USA) and performed multi-sequence analyses of human RXFP1, RXFP2 and relaxin proteins immunonogenic regions across different species: chimpanzee (*Pan Troglodyte*), cow (*Bos taurus*), dog (*Canis familiaris*), horse (*Equus cabalus*), and mouse (*Mus musculus*). These immunogenic protein sequences appeared to share approximately 99% identities across analyzed species. Based on this observation, we anticipated that the immunogenic sequences of human relaxin, RXFP1 and RXFP2 may also be conserved in the pig specie. All sequences were retrieved from the NCBI data repository for comparison using the T-Coffee version 7.71 alignments software.

#### Protein isolation and Western-immunoblot

Total proteins were extracted from frozen-thawed samples using the complete RIPA buffer (Santa Cruz Biotech Inc.). Equivalents of 20 μg total protein or whole lysates (of oocytes or cumulus cells) were resolved onto non denaturant and denaturant (SDS) PAGE gels and transferred to PVDF membranes (Millipore Corp, Bedford, USA). The specificities of anti-human RXFP1 and RXFP2 antibodies were verified on membranes pre-incubated overnight with or without their selective ligands (50 μg pRLN for RXFP1 and 10 μg INSL3 for RXFP2). The Fast Western Blot Kit containing the HRP-conjugated secondary antibody was used for immunodetection as recommended by the manufacturer (ECL Substrate, Piece). Briefly, membranes were exposed for 30 min to the primary antibodies (Relaxin, sc-20652; RXFP1, sc-50328; RXFP2, sc-50327; Santa Cruz Biotech Inc.) diluted at 1/500, then for 10 min to the secondary antibody diluted at 1/500 and followed by 2-3 min to the ECL reagent. Membranes were subsequently exposed to X-Ray film and results were digitalized for quantification (ImageJ, NIH Image).

#### In situ immunofluorescence

Samples were fixed in 4% methanol-free paraformaldehyde, permeabilized in 1% (v/v) Triton X-100, and blocked with 0.5% (v/v) normal goat serum. Samples were incubated overnight with or without antibodies (anti RXFP1 or 2; diluted 1:100), followed by 1h incubation with the FITC-conjugated secondary antibody (1:200). Samples were counterstained with the Hoechst dye and mounted onto slides for observation under a Zeiss Laser Scanning Microscope system (LSM510, Carl Zeiss Micro Imaging GmbH, Jena, Germany). Samples were washed three times with PBS/PVP-0.1% Tween 20 between steps.

### Statistical analysis

All experiments were repeated at least three times. Data were analyzed using a one-way analysis of variance (Systat Software, Inc.; Chicago, USA) followed by the Fisher's LSD test for pairwise comparisons. Expression level of RXFP1 and RXFP2 transcripts within the same sample type (cumulus cells, gametes or embryos) were compared using the Student's t-Test. Data are expressed as mean ± sd and p < 0.05 was considered significant.

## Results

### Detection of relaxin, RXFP1 and RXFP2 mRNA transcripts

**Figure 1:**
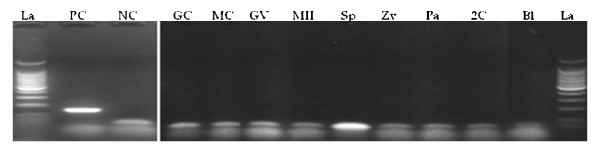
**Examination of relaxin mRNA expression in porcine gametes and embryos**. The RT-PCR shows relaxin mRNA expression in corpus lutea cells used as positive control (PC). There were no mRNA expression in granulosa cells (GC), mature cumulus cells (MC), immature (GV) and mature (MII) oocytes, spermatozoa (Sp), zygotes (Zy), parthenogenotes (Pa), cleaved embryos (2C) and blastocysts (Bl). Neonate porcine uteri mRNA were used as negative control (NC).

**Figure 2:**
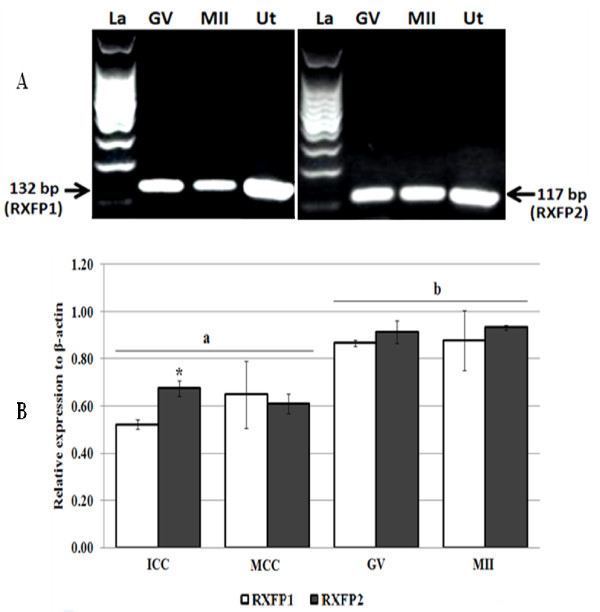
**Detection of RXFP1 and RXFP2 mRNA expression in porcine oocytes**. **A**: RT-PCR analyses show RXFP1 and RXFP2 mRNA expression in oocytes. Neonate porcine uteri mRNA were used as positive control (Ut). **B**: The quantitative PCR data revealed higher levels of both receptors mRNA in oocytes (GV and MII) compared to cumulus cells (ICC for immature and MCC for mature). Data represent a total of three independent replicates (mean ± sd). Significant differences between RXFP1 and RXFP2 within the ICC (*) and between oocytes and cumulus cells (a,b) (P < 0.05).

**Figure 3:**
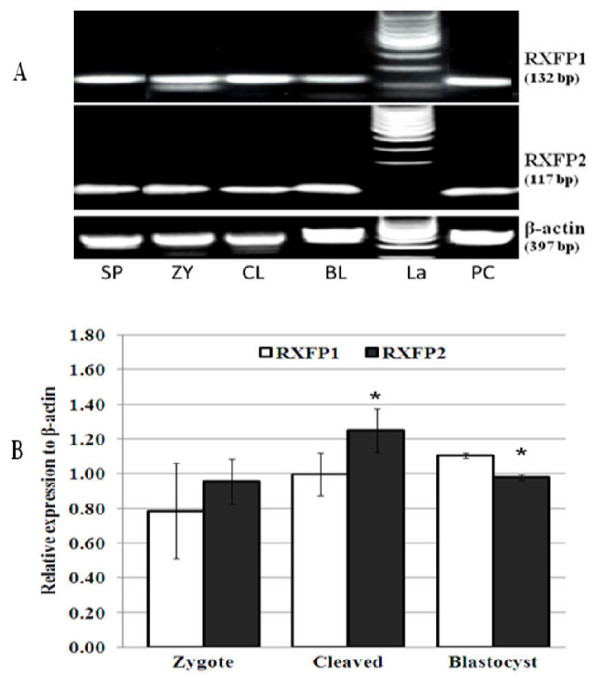
**Detection of RXFP1 and RXFP2 mRNA expression in porcine embryos**. **A**: RT-PCR analyses show RXFP1 and RXFP2 mRNA expression in spermatozoa (SP), zygote (ZY), cleaved embryos (2-8 cells; CL), and blastocysts (BL). **B**: The quantitative PCR data revealed significantly higher levels of RXFP2 and RXFP1 in cleaved embryos and blastocysts, respectively (*, P < 0.05). There was no significant difference in zygotes (P > 0.05). Neonate porcine uteri mRNA used as positive control (PC). (La) indicates the ladder. Data are mean ± sd of three independent replicates.

The product sizes generated by relaxin, RXFP1 and RXFP2 primers in corpus lutea and granulosa cells used as positive controls were conformed to their expected sizes of 164, 132 and 117 bp, respectively. We verified the amplification authenticities through PCR products sequencing followed by nucleotide sequence alignments. Sequencing electrophoregrams of relaxin, RXFP1 and RXFP2 revealed perfect matches with their reference sequences reported on NCBI (data not shown). We were not able to detect relaxin PCR products in all tested samples (Figure 1), while its receptor transcripts were found in immature (GV) and mature (MII) oocytes, and immature (ICC) and mature cumulus (MCC) cells (Figure 2A). Oocytes contained significantly higher levels than their cumulus cells, irrespective of the maturation time (GV vs. ICC and MII vs. MCC, p < 0.05; Figure 2B). Both receptor gene transcripts were detected in spermatozoa, zygotes, and embryos (Figure 3A). The levels of RXFP1 and RXFP2 mRNA were similar in the zygote. However, the RXFP1 levels appeared significantly lower in the cleaved-embryos and higher in blastocysts when compared to RXFP2 mRNA (Figure 3B; p < 0.05).

### Detection of relaxin, RXFP 1 and RXFP2 proteins

**Figure 4:**
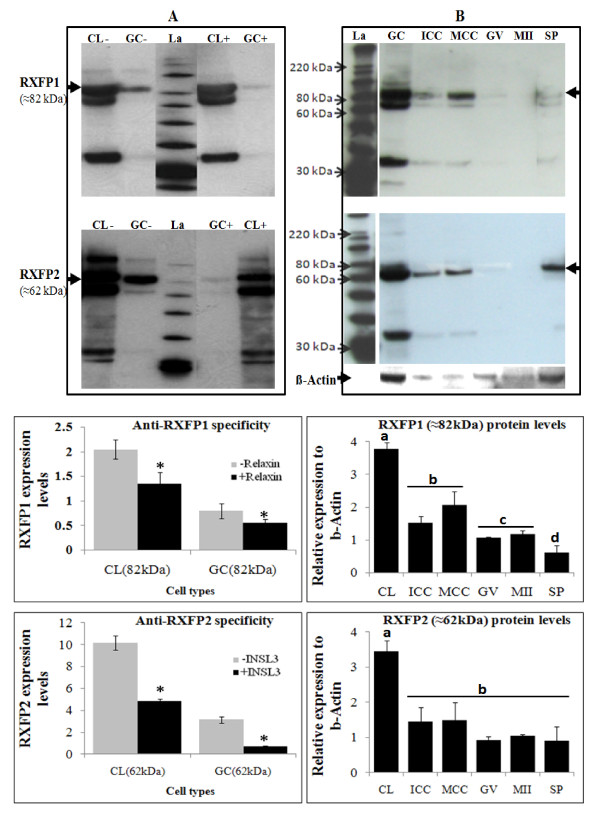
**Western-immunodetection of RXFP1 and RXFP2 protein**. The figure shows representative gel electrophoreses of at least three independent replicates. **A**: Validation of anti-human RXFP1 and RXFP2 in porcine ovarian granulosa cells (GC) and corpora lutea (CL). After gel electrophoreses, resolved proteins were transferred to PVDF membranes and before exposure to antibodies, membranes were pre-incubated (GC+ and CL+) or not (GC- and CL-) with RXFP1 or RXFP2 ligands (relaxin and INSL3, respectively). **B**: Membranes containing total proteins isolated from porcine granulosa cells (GC), immature (ICC) and mature (MCC) cumulus cells, immature (GV) and mature (MII) oocyte, and spermatozoa (SP) were incubated with anti-human RXFP1 or RXFP2 antibodies. Arrows indicate corresponding RXFP1 (≈82 kDa) and RXFP2 (≈62 kDa) bands. Band quantifications (using Image J) are presented in associated figures with statistic analyses of band intensities. Asterisks and letters indicate significant differences within the same (*) or between (a,b,c,d) cell types (P < 0.05; Student's t-Test).

**Figure 5:**
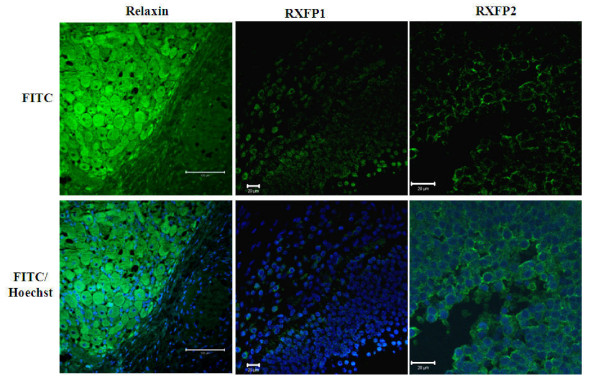
**Validation of antibodies on porcine tissues using immunofluorescence detection**. Validations were performed on a minimum of three slides prepared from three independent ovary collections. Sections of porcine ovaries were fixed for immunofluorescence detection of relaxin in corpora lutea and RXFP1 and RXFP2 in follicles. Micrographs indicate the labeling of protein targets (FITC, green color) in the luteal (relaxin) and granulosa and theca cells (RXFP1 and RXFP2). Both receptor signals are mostly located in the region of plasma membrane, while relaxin signal appears homogenous within the cytoplasm. Relaxin staining often delimits a dark hole corresponding to the nucleus stained with Hoechst dye (blue color). Not all cell types showed the FITC signals of protein targets. Scale bars = 100 μm.

**Figure 6:**
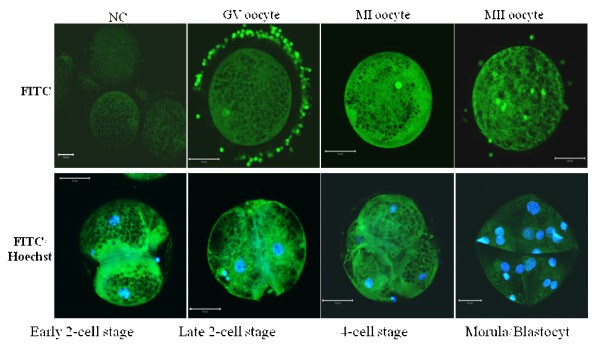
**Immunofluorescence detection of relaxin protein in porcine oocytes and embryos**. A minimum of 50 oocytes and embryos derived from 3 independent replicates were analyzed for immunofluorescence (IF). Micrographs show the IF detection (FITC, green color) of relaxin, which is homogenously distributed in oocytes and embryos. Nuclei are stained in blue with the Hoechst dye. The FITC signals in tested groups appear above the background as seen in the negative control group (NC). GV = Immature oocytes, MI = metaphase 1 oocytes, MII = metaphase II oocytes. Scale bars = 50 μm.

**Figure 7:**
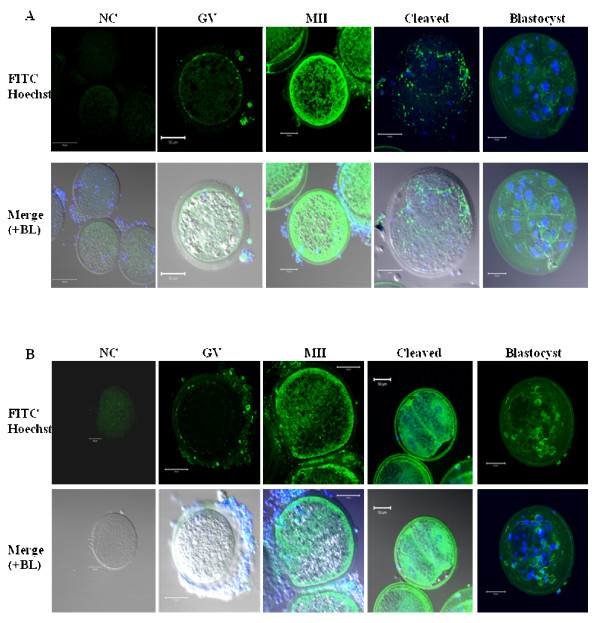
**Immunofluorescence detection of RXFP1 (A) and RXFP2 (B) protein in porcine oocytes and embryos**. A minimum of 50 oocytes and embryos derived from 3 independent replicates were analyzed for immunofluorescence (IF). Micrographs show the IF detection (FITC, green color) of RXFP1 (**A**) and RXFP2 (**B**). The plasma membrane location of both receptor signals is visible in oocytes, cumulus cells and blastocyst. The FITC signals in tested groups appear above the background as seen in the negative control group (NC). Nuclei are stained in blue with the Hoechst dye. GV = Immature oocytes, MII = metaphase II oocytes, BL = Bright light. Scale bars = 50 μm.

Ovarian cells and tissue sections known to express both relaxin and its receptors served as positive controls to confirm the specificity of antibodies. Using western-immunoblotting bands of approximately 82 and 67 kDa (RXFP1) and 78 and 62 kDa (RXFP2) were detected in corpora lutea (CL) and mural granulosa cells (GC) (Figure 4A). The specificity of antibodies was tested on membranes incubated with or without each receptor specific ligand (relaxin for RXFP1 and INSL3 for RXFP2) before their exposure to the corresponding antibody. All bands were notably reduced (Figure 4A). The 82 (RXFP1) and 62 kDa (RXFP2) bands were also detected in all experimental samples (GC, ICC, GV, MII and Spermatozoa; Figure 4B). Using *in situ *immunofluorescence, the presence of both relaxin and receptors was detected in corpus luteum and follicular cells, respectively; Figure 5). All tested samples (oocytes, cumulus cells, and embryos) also revealed the presence of relaxin (Figure 6), RXFP1 (Figure 7A) and RXFP2 (Figure 7B) protein receptors.

## Discussion

Here, we demonstrated that pig oocytes and embryos contain both relaxin and its receptors RXFP1 and RXFP2. Furthermore, timing and site of detections suggest putative roles of relaxin during oocyte maturation and embryo development. The primer pairs were effective for the detection of relaxin and its receptors expression in ovarian follicular and corpora lutea cells used as positive controls [12,18,25]. However, we were unable to detect the presence of relaxin messenger RNA in male and female gametes or developing embryos. But, its receptors were successfully detected in all samples. Expression levels of RXFP1 and 2 mRNA were unchanged in COCs during *in vitro *maturation, and oocytes maintained higher transcript levels than cumulus cells before and after maturation. Despite this observation, a likelihood of their involvement in oocyte maturation cannot be completely ruled out given a recent study reporting a beneficial role of relaxin receptors during maturation of rat oocytes [14]. In the male side, our findings include the pig to the growing list of species (i.e., rat, human) having RXFP1 and 2 receptor mRNA in their spermatozoa [26,27]. These RNA are likely remnants of transcriptional activities that occurred during spermatogenesis and might have roles during fertilization and beyond [28-30]. Indeed, we observed a differential abundance of both receptors at the embryo level, characterized by a significant accumulation of RXFP2 mRNA in cleaved embryos and RXFP1 mRNA in blastocysts. This developmental-stage accumulation may indicate a selective activation of these receptors in accordance with their specific signaling pathways [31]. Indeed, various reports have suggested the potential role of relaxin/receptor complex during implantation [32,33].

Furthermore, we analyzed the presence of relaxin and its receptors at the protein levels in both oocytes and embryos. Ovarian follicular and corpora lutea cells were used as positive controls as already reported in previous studies [11,15,16,25,34-36]. Here, we used anti-human relaxin and RXFP1/2, whose antigenic regions were found to be highly similar among various mammalians (cattle, chimpanzee, dog, horse, human, and mice). Western-immunoblotting revealed shorter molecular masses of both porcine receptors than their human and rat counterparts (82 kDa vs. 85-95 kDa for RXFP1, and 62 kDa vs. 80-90 kDa for RXFP2) [37,38]. Nevertheless, our results are still consistent with other reports using cells transfected with human RXFP1 [39] or RXFP2 [40]. Therefore, the above differences could be explained by the length or the differential glycosylation levels of RXFP1 and RXFP2 proteins between species [41-44]. In addition, the western-immunoblot revealed three isoforms of porcine RXFP1 and RXFP2, which may likely correspond to the mature (80 and 78 kDa; cell membrane delivery), immature or precursor (67 and 62 kDa; intracellular storage), and soluble (32 and 32 kDa; extracellular excretion) isoforms [39]. Both relaxin and receptor proteins were detected in all tested samples, indicating their involvement during porcine oocyte maturation, embryo development, and beyond [6,18,32-34,45-47]. It appeared that the oocyte relaxin protein content increased during maturation, probably due to transfers from the surrounding cumulus cells through gap junction communications [48]. Immunofluorescence (IF) staining allows the observation of differential accumulation of relaxin and receptor proteins between immature, metaphase I and metaphase II oocytes. This observation could explain the discrepancy between the WIB and IF results. Indeed, the WIB shows expression in pool of oocytes that are more likely at different maturational stage; not all oocytes placed in maturation reached the metaphase II stage by the moment of their collection. However, the IF data indicated that fully matured (metaphase II) oocytes contained the highest levels of proteins targeted, and therefore, suggesting their potential roles during oocyte maturation.

Protein detection in COCs appeared in opposition with their respective RNA expression data. It is likely that cumulus cells accumulate relaxin protein from the follicular fluid for a later transfer to the maturing oocyte. We observed that immature cumulus cells contained higher levels of relaxin than their corresponding oocytes. However, a possible existence of immature forms of relaxin not detected in the immature oocytes cannot be excluded. Regarding its receptor proteins, high levels were found in mature oocytes compared to immature ones that could be explained by either or both accumulations from cumulus cells during maturation and differential maturation stages of oocytes as mentioned above [48]. The detection of both protein systems during embryo development did not always reveal the expected cellular localization, which we attributed to transient modifications associated with cell divisions. In support our argument, the blastomeric perturbations of relaxin proteins systems distribution were reestablished more advanced embryonic stages such as the blastocysts. Additionally, the general and gradual decreased of relaxin/receptor protein systems throughout the embryo development may indicate beneficial effects that are limited during the early stages of the pre-implantation embryo development.

At the spermatozoa level, the detection of relaxin receptor proteins suggests their activation by an exposure to relaxin, whose beneficial effect on sperm motility has been reported in many species [29,30,49,50]. Nevertheless, it will be interesting to investigate whether RXFP2 (the most abundant), RXFP1, or both receptors are activated during fertilization.

## Conclusions

Our report provides, in our knowledge, the first evidence of porcine oocytes and pre-implantation embryos containing RXFP1 and RXFP2 receptors at both mRNA and protein levels. Both cell types do not express relaxin mRNA but seem to rely on an accumulation of relaxin protein that may occur during oogenesis. All together, the data indicate possible roles of the relaxin/receptors systems during oocyte maturation, embryo development, and beyond. Our study paves the way for further studies to (1) evaluate the specific involvement of each receptor type and (2) determine the effect of relaxin during fertilization and beyond. These additional investigations will bring more insights into the biological roles of relaxin/receptor complexes.

## Competing interests

The authors declare that they have no competing interests.

## Authors' contributions

JMF conceived and performed all the experiments, and wrote the first draft of the manuscript. JC R-M contributed in performing the experiments. STW and PLR contributed to the design of the study, revised and approved the manuscript. All authors read and approved the final draft.
